# Voraussetzungen für einen rationalen Antibiotikaeinsatz auf der Basis von Antibiotic Stewardship (ABS)

**DOI:** 10.1007/s00103-026-04224-8

**Published:** 2026-03-30

**Authors:** Katja de With, Rika Draenert, Dennis Nurjadi, Stefan Hagel, Ulrike Trost, Jan Fahrenkrog-Petersen, Winfried V. Kern, Evelyn Kramme

**Affiliations:** 1 Klinische Infektiologie, Institut für Infektiologie und Krankenhaushygiene, Universitätsklinikum Carl Gustav Carus an der TU Dresden, Fetscherstraße 74, 01307 Dresden, Deutschland; 2 Sektion Antimicrobial Stewardship (AMS), Deutsche Gesellschaft für Infektiologie e. V., Berlin, Deutschland; 3 Stabsstelle ABS, LMU Klinikum, München, Deutschland; 4 Akademie für Infektionsmedizin e. V., Berlin, Deutschland; 5 Institut für Medizinische Mikrobiologie, Universitätsklinikum Schleswig-Holstein, Campus Lübeck, Lübeck, Deutschland; 6 Institut f. Infektionsmedizin u. Krankenhaushygiene, Universitätsklinikum Jena, Jena, Deutschland; 7 Apotheke und Fächerverbund für Infektiologie, Pneumologie und Intensivmedizin, Charité – Universitätsmedizin, Campus Virchow-Klinikum, Berlin, Deutschland; 8 Apotheke, Pharmazeutische Logistik, Ambulanzversorgung, Charité – Universitätsmedizin Berlin, Campus Virchow-Klinikum, Berlin, Deutschland; 9 Ausschuss elektronische Verordnung, Bundesverband Deutscher Krankenhausapotheker (ADKA) e. V., Berlin, Deutschland; 10 Abteilung Infektiologie, Klinik für Innere Medizin II, Universitätsklinikum und Medizinische Fakultät Freiburg, Albert-Ludwigs-Universität, Freiburg im Breisgau, Deutschland; 11 Klinik für Infektiologie, Universitätsklinikum Schleswig-Holstein, Campus Lübeck, Lübeck, Deutschland

**Keywords:** Antimicrobial Stewardship, Antibiotikaresistenz, Diagnostic Stewardship, Antibiotikaverordnung, ABS-Team, Antimicrobial stewardship, Antimicrobial resistance, Diagnostic stewardship, Antibiotic prescribing, ABS team

## Abstract

Die rationale Antibiotikaverordnung ist angesichts des inadäquaten und übermäßigen Einsatzes von Antibiotika sowie der kritischen Resistenzentwicklung zu einer zentralen Priorität in der Humanmedizin geworden. In Deutschland ist bereits eine Reihe von Maßnahmen zur Optimierung des Antibiotikagebrauchs ergriffen worden. Hierzu zählen curriculare Antibiotic-Stewardship(ABS)-Fortbildungen zur Ausbildung zertifizierter ABS-Experten, die Erstellung einer S3-Leitlinie für ABS-Programme in Kliniken, der Aufbau regionaler ABS-Netzwerke mit Spezialisten aus Praxis, Pharmazie und Labor sowie geförderte Interventionsstudien in der ambulanten Versorgung. Signifikantes Verbesserungspotenzial liegt in der Etablierung und Finanzierung von ABS-Teams, infektiologischen Konsiliardiensten und Infektionsboards im Krankenhausbereich sowie in der Förderung innovativer mikrobiologischer Diagnostik und der Entwicklung nachhaltiger Konzepte für die ambulante Versorgung. Die systematische Integration mikrobiologischer und pharmazeutischer Expertise sowie die fortlaufende Implementierung und Weiterentwicklung moderner elektronischer Verordnungssysteme sind wichtige Bausteine. Sie können die Verordnungsqualität verbessern und unterstützen Schlüsselstrategien zur zielgerichteten Therapie und zur Verkürzung der Therapiedauer. Zur dauerhaften Etablierung dieser neuen Strukturen und ihrer Weiterentwicklung an intersektoralen Schnittstellen sind politische Unterstützung und umfassende Investitionen erforderlich.

**Zusatzmaterial online:**

Zusätzliche Informationen sind in der Online-Version dieses Artikels (10.1007/s00103-026-04224-8) enthalten.

## Abstract (en)

Given the inadequate and excessive use of antibiotics and the critical development of resistance, the rational prescription of antibiotics has become a key priority in human medicine. A broad range of initiatives to optimize antibiotic use has already been implemented in Germany. These include comprehensive antibiotic stewardship (ABS) training leading to the certification of ABS specialists; the development of a guideline for ABS programs in hospitals; the establishment of regional ABS networks integrating expertise from clinical practice, pharmacy, and microbiology; and the execution of funded interventional studies within the outpatient sector. There is significant potential for improvement in the establishment and financing of ABS teams, infectious disease consultation services, and infection boards in hospitals as well as in the promotion of innovative microbiological diagnostics and the development of sustainable concepts for outpatient care. The systematic integration of microbiological and pharmaceutical expertise into ABS programs is essential, as is the ongoing implementation and further development of modern electronic prescription systems. All these measures substantially improve prescription quality and support key strategies for targeted therapy and shorter treatment durations. To ensure the long-term establishment of these new structures and their further development at intersectoral interfaces, political support and substantial investment are indispensable.

## Hintergrund

Es gibt gute Evidenz für den Zusammenhang zwischen intensivem Antibiotikaeinsatz in der Humanmedizin und ansteigenden bakteriellen Resistenzraten [1]. Die Zusammenhänge zwischen Verordnung/Anwendung, Selektionsdruck, Resistenzentwicklung und -ausbreitung sind sehr komplex. Resistenzen können unterschiedlich schnell entstehen und sich ausbreiten. Ihre Beständigkeit kann von vorübergehend bis irreversibel variieren. Co-Selektion kann bewirken, dass Wirkstoff X Resistenzen gegen chemisch nicht verwandtes Y und mitunter Z erzeugt. Dabei führt das Absetzen von X paradoxerweise nicht zu einer Reduktion, sondern (besonders bei weiterer Gabe von Y oder Z) zu einer Verstärkung der Resistenzen gegenüber X [2]. Für die Beurteilung humanmedizinischer Maßnahmen ist deshalb auch der erhebliche Antibiotikaeinsatz außerhalb der Humanmedizin, besonders in der Tierhaltung, relevant. Er fördert den Selektionsdruck und somit das Infektionsrisiko des Menschen durch resistente Bakterien. Der Antibiotikaeinsatz in der Humanmedizin weist erheblichen Verbesserungsbedarf auf. Dieser betrifft Indikationsstellung, Wirkstoffauswahl und -dosierung, Therapiedauer sowie die Notwendigkeit zur Deeskalation und Umstellung von parenteraler auf orale Therapie [3–5]. Antibiotic Stewardship (ABS; syn. Antimicrobial Stewardship – AMS – oder Antimicrobial Stewardship Programme – ASP) ist eine Reaktion auf diese suboptimale Verordnungsqualität in der Humanmedizin – bei zugleich zunehmenden Antibiotikaresistenzen und weniger Antibiotikaneuentwicklungen und Infektionsbehandlungsalternativen. ABS wurde prägnant beschrieben als „programmatisches, nachhaltiges Bemühen einer medizinischen Institution“ (oder eines Gesundheitssystems) „um Verbesserung und Sicherstellung einer rationalen Antiinfektiva-Verordnung“ [5]. Eine Verlangsamung der Resistenzentwicklung ist ein wichtiges Ziel. Zudem sollen die Behandlungsergebnisse optimiert, Ressourcen geschont und unnötige Toxizität bzw. Effekte vermieden werden. Unerwünschte indirekte Effekte sind für Allergien und auf das Mikrobiom beschrieben. Mikrobiomveränderungen wurden zudem mit verschiedenen nichtinfektiösen Erkrankungen, darunter Übergewicht, in Verbindung gebracht. In einer Vielzahl an Publikationen wurden ABS-Programme als kosteneffektiv bewertet [6]. In dem vorliegenden Übersichtsartikel sollen die Entwicklung und Auswirkungen von ABS-Aktivitäten in Deutschland sowie ausgewählte aktuelle Strategien für eine Therapieoptimierung dargestellt werden.

## Entwicklung von „ABS“ in Deutschland

In vielen Ländern sind inzwischen ABS-Aktivitäten auf verschiedenen Ebenen entwickelt worden [7]. Auf der Versorgungsebene sind es Information und Schulung, (lokale) Leitlinienanpassung und -adhärenz, Benchmark-Systeme und interne Qualitätssicherung. National erfordern derartige Programme und Initiativen die Auseinandersetzung mit ärztlicher Aus- und Weiterbildung, Berufspolitik, der Vergütung von Arzt- und Laborleistungen und der Förderung von klinischer und Versorgungsforschung. Zusätzlich werden Strukturen zur Erstellung von Leitlinien sowie Implementierungshilfen und Qualitätssicherungsinstrumente in der Infektionsmedizin benötigt. Welche wesentlichen Entwicklungen sind hierzu in Deutschland zu beobachten?

### ABS-Fortbildungsinitiative für Krankenhausärzte und Apotheker.

**Abb. 1:**
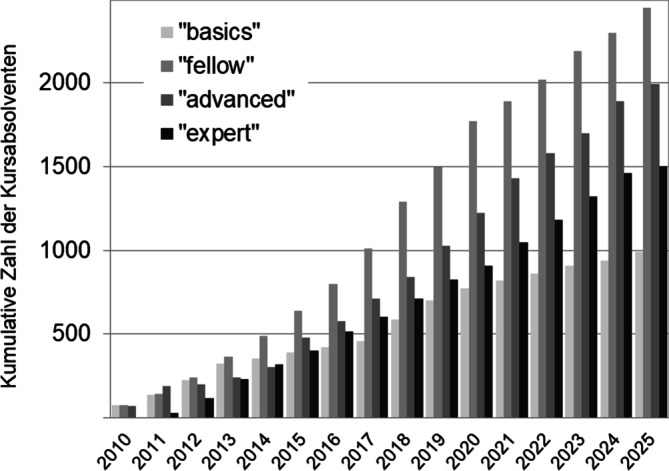
Absolventen der Antibiotic-Stewardship(ABS)-Kurse der Deutschen Gesellschaft für Infektiologie (DGI) im zeitlichen Verlauf. Angezeigt sind die kumulativen Zahlen der Teilnehmerzahlen an den verschiedenen Fortbildungsmodulen. Quelle: eigene Daten

Unter den Bedingungen fehlender fachärztlicher Expertise und notwendiger Wissensvermittlung zum Thema rationale Antiinfektivatherapie wurde 2010 die ABS-Fortbildungsinitiative gestartet. Ziel war es, mittels einer curricularen Fortbildung (4 aufeinander aufbauende Module plus eine eigene Praktikumsarbeit in der entsendenden Klinik) die Expertise vor allem in kleineren Kliniken zu steigern, ohne neues Personal einstellen zu müssen. In der ersten Phase der Fortbildungsinitiative (2010 bis 2014) wurde das von der Universität Freiburg betreute Projekt vom Bundesministerium für Gesundheit finanziell bezuschusst. Zentral war die Erkenntnis, dass nicht nur großer Bedarf bestand, sondern auch ausgeprägte Bereitschaft der Interessenten, Zeit und Geld zu investieren. Im Verlauf der Jahre wurde die Initiative ideell von der Sektion AMS der Deutschen Gesellschaft für Infektiologie (DGI) unterstützt. Organisatorisch wurde sie in der eigens gegründeten DGI-Fortbildungsakademie (Akademie für Infektionsmedizin) verankert und weiterentwickelt. Allein hierüber wurden seither rund 1500 sogenannte ABS-Experten fortgebildet und seitens der DGI zertifiziert (Abb. 1). Weitere Institutionen, darunter einige Landesärztekammern, haben die Fortbildung (teils oder vollständig) nach dem Mustercurriculum der Bundesärztekammer eingeführt und bieten diese bis heute an. Wir schätzen, dass die Zahl der Absolventen damit insgesamt bei 3000 liegen dürfte. Der berufliche Hintergrund der Absolventen ist überwiegend die Innere Medizin und Intensivmedizin/Anästhesie (Tab. 1).

**Tab. 1:** Beruflicher Hintergrund von Antibiotic-Stewardship(ABS)-Experten (bezogen auf die Tätigkeit zum Zeitpunkt der Kursteilnahme; ohne Mehrfachqualifikationen; nur Absolventen von Kursen der Deutschen Gesellschaft für Infektiologie – DGI). Quelle: eigene Daten.

Innere Medizin	33 %
Anästhesiologie	21 %
Klinische Pharmazie	13 %
Medizinische Mikrobiologie	11 %
Krankenhaushygiene	5 %
Sonstige	17 %

### ABS-Leitlinie in Akutkrankenhäusern und Umsetzung.

Begleitend zur Fortbildungsinitiative wurden seitens der Universität Freiburg und der DGI-Sektion AMS eine S3-Leitlinie zu ABS im Akutkrankenhaus, ein Antibiotikaverbrauchsmonitoring in Kooperation mit dem Bundesverband Deutscher Krankenhausapotheker – Modell für die spätere Entwicklung der „Antibiotikaverbrauchssurveillance“ (AVS) des Robert Koch-Institutes (RKI) – und die Diskussion über ABS-Qualitätsindikatoren im Krankenhaus initiiert [8].

Die aktuell in Überarbeitung befindliche ABS-Leitlinie empfiehlt für Akutkrankenhäuser ein interdisziplinäres ABS-Team mit einer Mindestausstattung von 1 Vollzeitäquivalent (VZÄ) pro 500 Betten und mit einem Mandat seitens Klinikleitung und Ressourcen (Tab. 2). Ungeachtet dieser Empfehlungen, die sich mit internationalen Leitlinien decken, beeinträchtigen im deutschen Akutklinikbereich strukturelle Defizite weiterhin die notwendige Qualitätsverbesserung des Infektionsmanagements und der Antibiotikaverordnung. Umfragen und der Vergleich mit internationalen Publikationen bestätigen, dass es in erster Linie an Personalfreistellungen fehlt [9]. Zudem gibt es in den Kliniken für den Bereich rationale Antiinfektivaverordnung kaum übergeordnete Verantwortlichkeiten, obwohl inzwischen zahlenmäßig (bezüglich der Mindestausstattung 1:500) ABS-fortgebildetes Personal ausreichend zur Verfügung steht. Darüber hinaus haben mittlerweile zahlreiche Ärzte die Facharzt- oder Zusatzweiterbildung Infektiologie erworben [3, 4]. Neben zu geringer Personalkapazität stellen hohe Fluktuation, zu geringe Bereitschaft zum interdisziplinären Arbeiten und fehlende Anerkennung große Hindernisse bei der Umsetzung von ABS-Maßnahmen vor Ort dar [10]. Die neu gestartete externe Sepsis-Qualitätssicherung eröffnet möglicherweise die Chance, strukturelle Defizite zu adressieren. Hier werden analog der ABS-Leitlinie ABS-Teams verlangt, die die lokalen Leitlinien erarbeiten, aktualisieren und Hilfestellungen zur Adhärenz anbieten sollen.

**Tab. 2:** Empfohlene Ausstattung der Antibiotic-Stewardship-(ABS-)Teams für Akutkrankenhäuser entsprechend der deutsch-österreichischen S3-Leitlinie.

Kliniktyp/-größe^a^ und Schwerpunkte^b^	Mindestbedarf (1 VZÄ pro 500 Betten)	Zusatzbedarf (0,5 VZÄ je definiertem Schwerpunkt)	Gesamtbedarf (VZÄ)
Universitätsklinikum, 1200 Betten mit Organtransplantationseinheit, Hämatologie inkl. allogener Stammzelltransplantation, mit Fachabteilung für Kinder- und Jugendmedizin inkl. Neonatologie, und eigenen Fachabteilungen für Herzchirurgie und für Neurochirurgie, 5 Intensivstationen	2,4	2,4	4,8
Großes Allgemeinkrankenhaus, 700 Betten, mit Fachabteilung für Kinder- und Jugendmedizin inkl. Neonatologie, und eigenen Fachabteilungen für Neurochirurgie, Herzchirurgie und Orthopädie mit Schwerpunkt Gelenkersatz	1,4	1,0	2,4
Mittleres Allgemeinkrankenhaus, 450 Betten, mit Fachabteilung für Orthopädie mit Schwerpunkt Gelenkersatz	0,9	0,2	1,1
Kleines Allgemeinkrankenhaus, 250 Betten, ohne Schwerpunkte	0,5	–	0,5

Abk.: *VZÄ* Vollzeitäquivalent

^a^ohne Berücksichtigung von psychiatrischen, psychotherapeutischen/psychosomatischen, nuklearmedizinischen und Rehabilitationsbetten

^b^Schwerpunkte können sein: Organtransplantationszentrum bzw. -einheiten, Fachabteilung/Klinik für Hämatologie inkl. allogener Stammzelltransplantation; Fachabteilung für Kinder- und Jugendmedizin inkl. Neonatologie; Schwerpunkt im Bereich orthopädischer Gelenkersatz und/oder Herzchirurgie und/oder Neurochirurgie (wenn nur eine (0,2 VZÄ) oder 2 (0,4 VZÄ) der Disziplinen vorhanden sind, entsprechende Reduktion); Kliniken mit mehr als 4 intensivmedizinischen Behandlungseinheiten/Stationen oder mehr als 50 Intensivbetten oder einer eigenen (bettenführenden) Fachabteilung Intensivmedizin

### ABS-Team, infektiologisches Konsil, Infektionsboards.

Die Hauptaufgabe der ABS-Teams liegt in der krankenhausweiten Konzeption und Implementierung maßgeschneiderter ABS-Programme für die häufigsten Infektionen. Für komplexe Erkrankungen, Infektionen durch seltene Erreger oder mit atypischen Verläufen ist das infektiologische Konsil unverzichtbar. Es bietet spezialisierte Expertise zur Berücksichtigung von Differenzialdiagnosen, Optimierung der Diagnostik, Management und Antiinfektivatherapie. Studien zeigen, dass solche Konsile zu signifikanter Reduktion unangemessener Antibiotikaanwendungen, verkürzter Krankenhausverweildauer und einer verbesserten Patientensicherheit führen können; auch scheint nach einigen Untersuchungen dadurch das Überleben verbessert [11, 12]. Für bestimmte Infektionskrankheiten, wie z. B. Endokarditis, Gelenkprotheseninfektionen oder Sepsis, wird heute zusätzlich eine formalisierte, multidisziplinäre Entscheidungsfindung mit allen beteiligten Behandlern in sogenannten Boards empfohlen. Hier sollen insbesondere Fragen zu weiterführender Diagnostik, differenzierter operativer oder konservativer Therapie sowie zur Nachsorge im Konsens beantwortet werden. Sie dienen auch als interne Fortbildungsplattform, indem klinische Fälle anhand aktueller Evidenz gemeinsam erörtert werden. Eine strategisch verknüpfte Infrastruktur aus ABS-Programmen, infektiologischen Konsiliardiensten und interdisziplinären Boards kann den Behandlungserfolg des einzelnen Patienten optimieren und zu einem zukunftsfähigen Infektionsmanagement im Krankenhaus beitragen.

### Studien zur ambulanten Antibiotikaverordnungsqualität.

Für den ambulanten Sektor in Deutschland sind bisher außerhalb von Studien keine landesweiten nachhaltigen Fortbildungs- und Schulungsaktivitäten im Bereich ABS entstanden. Eine Reihe von Studien hat sich mit Möglichkeiten der Verbesserung der Antibiotikaverordnungsqualität in Deutschland beschäftigt. Multimodale Konzepte in allgemeinärztlichen Praxen hatten die Reduktion unnötiger Verordnungen bzw. die Vermeidung unnötig breit wirksamer Wirkstoffe zum Ziel. Bewährt hat sich neben Schulungen und „Audit & Feedback“ (Rückmeldung der eigenen Verbrauchsdaten im Vergleich) eine verbesserte Arzt-Patienten-Kommunikation als wichtige Interventionskomponente [13–15]. In der „ARena“-Studie (überwiegend in Bayern durchgeführt; Projektlaufzeit 01/2017 bis 09/2020) wurde in der Behandlung unkomplizierter Infektionen ein signifikanter Rückgang der Antibiotikaverordnungsraten gezeigt. Sowohl für Ärzte als auch für die Öffentlichkeit wurden Qualitätszirkel, Fortbildung über E‑Learning, Information über das eigene Verordnungsverhalten im Vergleich, kontaktabhängige und ergebnisabhängige Vergütung sowie zielgruppenadaptierte Patienteninformationen angeboten [16]. Die Effekte in anderen Studien (z. B. „CHANGE-3“, „RESIST“ und „ELEKTRA“) waren eher schwach und teilweise nicht signifikant [17–20]. Eine deutlich geringere Reduktion von Nichterstlinienpräparaten (− 13 %) bei Harnwegsinfektionen wurde in der „RedAres“-Studie (überregional; Projektlaufzeit 04/2021 bis 03/2022) gezeigt – bei einer Reduktion der Gesamtverordnungsrate um 8 %. Die Intervention war hier die Schulung zu aktuellen Leitlinienempfehlungen, „Audit & Feedback“ und Information über die regionale Resistenzsituation (über Daten des RKI; [21, 22]).

### ABS-Netzwerke.

Die oben beschriebenen Projekte sind kontrollierte Interventionsstudien, deren weitere Förderung und damit Fortführung bzw. breitere Umsetzung in die Praxis bislang unklar ist. Außerdem bestehen seit Längerem regionale Initiativen, die unter dem Begriff ABS-Netzwerke zusammengefasst werden. Das bekannteste ambulante Netzwerk ist AnTiB („Antibiotische Therapie in Bielefeld“), das 2016 vom Ärztenetz Bielefeld initiiert wurde [23]. Partner sind zudem Krankenhäuser und Labore in der Region. Obwohl das Netzwerk nicht primär forschungsorientiert ist, sind in Kooperation mit der Fakultät für Gesundheitswissenschaften der Universität Bielefeld versorgungswissenschaftliche Auswertungen hervorgegangen [24, 25]. Bei einer der Auswertungen der Antibiotikaverordnungen von Kinderarztpraxen zwischen 2015 und 2018 ist der sehr niedrige Verbrauch von Cefuroxim-Axetil auffällig [25].

Als Teil des ABS-Netzwerks Bielefeld/Ostwestfalen-Lippe konzentriert sich AnTiB auf die verbindliche Etablierung lokaler ambulanter Therapieempfehlungen für Praxen und Notfallpraxen. Diese werden unter Einbezug ambulanter/stationärer Schnittstellen und infektiologischer Fachgesellschaften entwickelt. Das Netzwerk bietet zudem Fortbildungen (inkl. Qualitätszirkel) an und ermöglicht Rückmeldungen zum Antibiotikaverbrauch über die Kassenärztliche Vereinigung. Es wurde inzwischen zum ABS-Netzwerk Westfalen-Lippe ausgebaut und deckt größere Gebiete unter Einbindung weiterer Kliniken wie den Universitätskliniken Bochum und Münster ab [26]. Weitere aktive ABS-Netzwerke bestehen in anderen Regionen (Infobox). Das Netzwerk Westfalen-Lippe ist insofern einzigartig, als es primär von der niedergelassenen Ärzteschaft ausgeht und getragen wird, während die übrigen ABS-Netzwerke ausschließlich oder überwiegend Klinikkooperationen sind. Davon abzugrenzen sind regionale Multiresistente-Erreger-Netzwerke (MRE; „multidrug-resistant organisms“ – MDRO), die unter Federführung des öffentlichen Gesundheitsdienstes vorrangig Hygienefragen bearbeiten und praktische Hilfen zum Hygienemanagement bei Patienten mit MRE entwickeln. Sie sind in vielen Regionen formal etabliert und arbeiten mit zahlreichen Kooperationspartnern.

## Änderungen der Antibiotikaverordnungsmenge

**Abb. 2:**
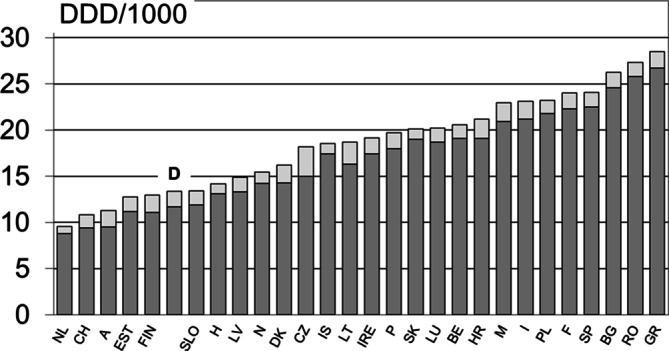
Antibiotikaverordnungsdichte im ambulanten und stationären Bereich (definierte Tagesdosis/„defined daily dose“ – DDD – pro 1000 Einwohner bzw. Versicherte der gesetzlichen Krankenkassen – GKV – und Tag) im europäischen Vergleich (Daten für 2023). Quelle: European Centre for Disease Prevention and Control (ECDC) 2024, Bundesamt für Gesundheit (BAG, Schweiz) 2025, Länderlegende: *NL* Niederlande; *CH* Schweiz; *A* Österreich; *EST* Estland; *FIN* Finnland; *D* Deutschland, *SLO* Slowenien; *H* Ungarn; *LV* Lettland; *N* Norwegen; *DK* Dänemark; *CZ* Tschechien; *IS* Island; *LT* Litauen; *IRE* Irland; *P* Portugal; *SK* Slowakei; *LUX* Luxemburg; *BE* Belgien; *HR* Kroatien; *M* Malta; *I* Italien; *PL* Polen; *F* Frankreich; *SP* Spanien; *BG* Bulgarien; *RO* Rumänien; *GR* Griechenland

Welche wesentlichen Änderungen gibt es auf der Ebene der Antibiotikaverordnung? Deutschland zählt nicht zu den Hochverbraucherregionen [27–29]. Über alle Wirkstoffklassen hinweg und auf die gesamte Bevölkerung bezogen, liegt die geschätzte Verbrauchsdichte in der ambulanten Versorgung im unteren europäischen Drittel (Abb. 2), im stationären Bereich im Mittelfeld. Im Vergleich mit anderen Niedrigverbraucherländern in Europa ist in Deutschland der relativ hohe Anteil von Oralcephalosporinen eine Besonderheit. Cefuroxim-Axetil hat in den 20 Jahren bis 2016 maßgeblich durch einen nahezu 10-fachen Anstieg der ambulant verordneten Tagesdosen dazu beigetragen [3, 30].

**Abb. 3:**
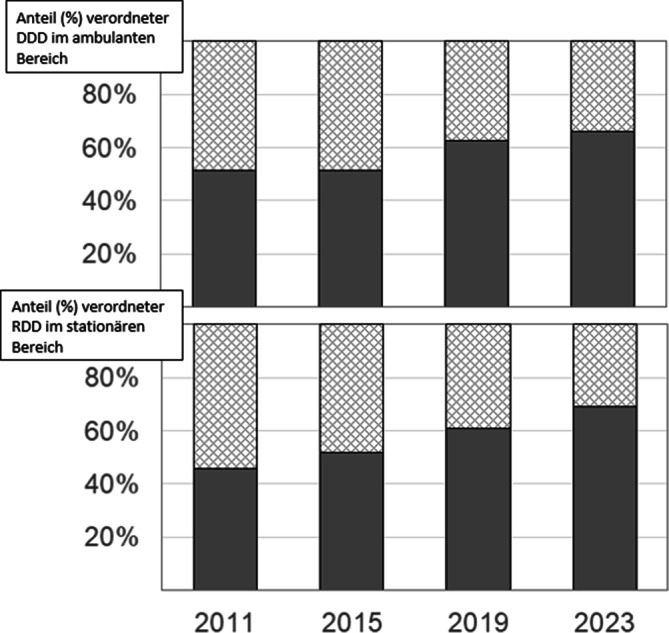
Entwicklung der Relation Penicillin-Derivate (grau) zu Cephalosporinen (schraffiert) bezogen auf verordnete Tagesdosen im ambulanten (oben) und stationären (unten) Bereich zwischen 2011 und 2023. Ambulant: definierte Tagesdosen (*DDD* „defined daily dose“), stationär: adaptierte Tagesdosen für Krankenhausbehandlungen (*RDD* „recommended daily dose“) gemäß Surveillance-Projekt des Bundesverbands Deutscher Krankenhausapotheker (ADKA), der Abteilung für Infektiologie am Universitätsklinikum Freiburg (IF) und der Deutschen Gesellschaft für Infektiologie (DGI). Quelle: European Surveillance of Antimicrobial Consumption Network (ESAC-Net)

**Abb. 4:**
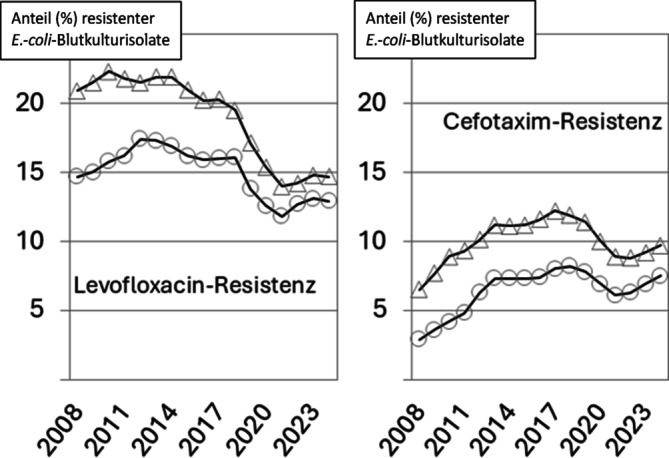
Anteil (%) resistenter *E. coli*-Blutkulturisolate gegenüber Fluorchinolonen (Levofloxacin) bzw. Drittgenerationscephalosporinen (Cefotaxim) im ambulanten (О) und stationären (∆) Sektor. Quelle: Antibiotika-Resistenz-Surveillance (ARS), Robert Koch-Institut (RKI)

In den letzten Jahren sind einige günstige Entwicklungen zu beobachten. Vor allem bei Kindern werden weniger Antibiotika eingesetzt [31]. Die inadäquate und extrem hohe Verordnungsrate von Oralcephalosporinen im ambulanten Sektor (Abb. 3) und die Fluorchinolon-Verordnungen ambulant und stationär wurden korrigiert. Im stationären Bereich werden zudem – anders als noch vor 10 bis 20 Jahren – mehr Penicilline als Cephalosporine eingesetzt (Abb. 3; [32]). Diese Änderungen gehen zumindest mit dem Rückgang der Resistenzen gegenüber Fluorchinolonen und einer Stabilisierung der Resistenzen gegenüber Cephalosporinen einher (Abb. 4). Auch die veränderte Epidemiologie der *C. difficile*-Infektionen kann mit dem reduzierten Einsatz der Fluorchinolone erklärt werden [33].

Eine weitere Reduktion des ambulanten Antibiotikaverbrauchs wird zunehmend schwierig. Die Verordnungsraten sind bereits relativ niedrig und die sinkenden stationären Behandlungskapazitäten wirken sich gegenläufig aus. Die Umsetzungsfähigkeit der Praxen, weitere Instrumente einzusetzen, ist begrenzt. Eine Verbesserung wäre nur durch Investitionen zu erreichen. Intersektoral organisierte regionale ABS-Netzwerke könnten die Bewältigung der wachsenden Schnittstellenprobleme unterstützen, die sich aus der zunehmend abgestuften stationären Versorgung und der weiteren Bettenreduktion ergibt. Quantitative Verbrauchsziele alleine sind nicht ausreichend. Sie müssen durch qualitative Ziele und aussagekräftige Qualitätsindikatoren ergänzt werden. Dies erfordert eine deutlich höhere Investitionsbereitschaft [3, 4, 19, 34, 35].

## Diagnostik für eine zeitnahe und zielgerichtete Therapieanpassung

Präzise, zügige und sorgfältig ausgewählte mikrobiologische Diagnostik ist ein wichtiger Baustein erfolgreicher ABS-Programme. Technologische Fortschritte ermöglichen schnellere Therapieentscheidungen, deren nachhaltige Wirkung jedoch erst durch die konsequente Integration in strukturierte ABS-Prozesse mittels zeitnaher Kommunikation, selektiven Reportings und interdisziplinärer Zusammenarbeit erreicht wird [36].

### Blutkulturdiagnostik.

Die rasche Identifikation bakterieller Erreger aus positiven Blutkulturen stellt eine entscheidende Voraussetzung zur zeitnahen Optimierung antimikrobieller Therapien dar [37]. Die Zeit bis zum Therapiebeginn ist dabei ein entscheidender Faktor für das Behandlungsergebnis [38–40]. Moderne Technologien wie die Matrix-unterstützte Laserdesorptions‑/Ionisations-Time-of-Flight-Massenspektrometrie (MALDI-TOF MS) haben die mikrobiologische Diagnostik grundlegend verändert [41]. Dieses Verfahren ermöglicht die Identifizierung von Bakterien und Hefen innerhalb von 15–30 min nach einer positiven Blutkultur [41, 42].

Trotz dieser erheblichen Beschleunigung bleibt die MALDI-TOF-basierte Erregeridentifikation kulturbasiert und damit vom Wachstum von Mikroorganismen in der Blutkultur abhängig. Eine direkte Anwendung auf Primärmaterial (z. B. Vollblut) ist bislang nur eingeschränkt möglich, da die mikrobielle Last häufig unter der Nachweisgrenze liegt. Zudem liefert MALDI-TOF keine Resistenzinformationen, weshalb für eine vollständige Befundung weiterhin ein nachgeschalteter Empfindlichkeitstest erforderlich ist.

Ergänzend zur kulturbasierten MALDI-TOF-Analytik stehen inzwischen molekulare Schnelltestsysteme zur Verfügung, die eine noch frühere Identifikation ermöglichen (siehe Onlinematerial 1 [36, 43–62]) [63, 64]. Kommerzielle Multiplex-PCR(Polymerasekettenreaktion)-Plattformen erlauben die gleichzeitige Detektion von bis zu 40 mikrobiellen Erregern und klinisch relevanten Resistenzgenen (z. B. *mecA, blaCTX‑M, vanA/B*). Sie liefern innerhalb von etwa 60–90 min nach einer positiven Blutkultur klinisch verwertbare Ergebnisse [36, 64]. Dadurch kann der Zeitraum bis zur gezielten Therapie entscheidend verkürzt werden. Voraussetzung dafür ist die Integration in ein funktionierendes ABS-Team. Aufgrund hoher Anschaffungs- und Betriebskosten kommen die Systeme bisher jedoch vor allem in zentralisierten mikrobiologischen Laboren oder spezialisierten Einrichtungen zum Einsatz.

### Rapid Antimicrobial Susceptibility Testing.

Ein Beispiel für eine kostengünstigere und schnelle phänotypische Resistenztestung ist das vom European Committee on Antimicrobial Susceptibility Testing (EUCAST) entwickelte Rapid-Antimicrobial-Susceptibility-Testing-(RAST-)Verfahren [65, 66], welches sich ohne großen technischen Aufwand in die mikrobiologische Routinediagnostik integrieren lässt [67]. Das Verfahren basiert vollständig auf der klassischen Agardiffusionstechnik. Unter Routinebedingungen konnte gezeigt werden, dass bereits nach 4–6 h Inkubation verlässliche vorläufige Empfindlichkeitsdaten vorliegen, die eine frühzeitige Anpassung der antimikrobiellen Therapie ermöglichen. In einer multizentrischen Studie mit über 2000 klinischen Isolaten (vorwiegend *E. coli, K. pneumoniae, P. aeruginosa* und *S. aureus*) wurde eine Übereinstimmung von mehr als 90 % zwischen den vorläufigen RAST-Kategorisierungen (S/I/R: „susceptible/susceptible, increased exposure/resistant“), welche nach 4–6 h vorlagen, und den endgültigen Testergebnissen des EUCAST-Standardverfahrens, welche erst nach 16–20 h vorlagen, nachgewiesen [68]. Damit liefert das EUCAST-RAST-Verfahren reproduzierbare und klinisch verwertbare Resistenzinformationen noch am selben Tag, sodass die Behandler bereits in der Frühphase einer Sepsis eine gezielte antimikrobielle Therapie einleiten können [62, 69]. Entsprechend zeigte eine Studie an Patienten mit Bakteriämien durch gramnegative Erreger, dass die Nutzung neuer diagnostischer Verfahren zur schnellen Erregeridentifizierung, inkl. Empfindlichkeitstestung, die Dauer der intravenösen Antibiotikatherapie im Median um 3,5 Tage und die Krankenhausverweildauer um fast 2 Tage signifikant reduzierte [70]. Eine Metaanalyse bestätigte, dass nur die Kombination aus Rapid Diagnostic Testing und aktivem ABS-Programm mit einer signifikanten Reduktion der Sterblichkeit assoziiert war [71].

### Selektives Reporting.

Dem mikrobiologischen Labor kommt in der Befundgestaltung eine relevante Bedeutung in der Therapiesteuerung zu [72]. Beispiele hierfür liefern Studien zu Harnwegsinfektionen, welche den Einfluss der Empfindlichkeitsberichterstattung auf das Verordnungsverhalten untersuchten. In einer kanadischen Kohortenstudie wurde ein Antibiotikum signifikant (3-fach) häufiger verschrieben, wenn es im Befundbericht genannt wurde [73]. Eine französische Arbeit konnte durch selektive Antibiogrammberichtung den Anteil verordneter 3. Generations-Cephalosporine um ca. 9 % senken [74]. Erfahrungen mit selektiver Antibiogrammberichtung zur Therapiesteuerung gibt es zu *S.-aureus*- bzw. *E.-faecalis*-Bakteriämien auch aus Deutschland [75, 76]. Obwohl viele Studien diesen Ansatz als ergänzende Maßnahme vorgeschlagen haben, gibt es bislang zu wenige Untersuchungen, die den Einfluss der selektiven Berichterstattung auf die Prävalenz von Resistenzen belegen.

## Ausgewählte Strategien zur Therapieoptimierung

### Pharmakokinetik und Pharmakodynamik.

Für die optimale Dosierung von Antiinfektiva sind deren pharmakokinetische (PK) und pharmakodynamische (PD) Eigenschaften unter Berücksichtigung der klinischen Situation und der minimalen Hemmkonzentration des Erregers entscheidend.

Bei Betalaktamen ist die Dauer, während der die freie Wirkstoffkonzentration oberhalb der minimalen Hemmkonzentration liegt, der zentrale Wirksamkeitsparameter. In einer Studie mit > 7000 Sepsis-Patienten war die kontinuierliche Infusion (ohne therapeutisches Drug-Monitoring, TDM) von Piperacillin/Tazobactam oder Meropenem einer 30-minütigen Kurzinfusion hinsichtlich klinischer Heilungsrate überlegen und mit einer tendenziell geringeren 90-Tage-Sterblichkeit assoziiert [77]. Eine begleitende Metaanalyse ergab eine signifikante Letalitätsreduktion [78]. In nahezu allen eingeschlossenen Studien wurde eine kontinuierliche und keine prolongierte Infusion verwendet. Entsprechend empfiehlt die EUCAST bei als „I“ klassifizierten Erregern für ausgewählte Betalaktame eine Dosiserhöhung und/oder eine verlängerte Infusion über 3–4 h [79].

Zur raschen Erreichung therapeutischer Spiegel sollte zu Therapiebeginn bei kritisch kranken Patienten eine initiale Bolusgabe erfolgen, als zusätzliche halbe Dosis oder durch schnelle Infusion der ersten Hälfte der Gesamtdosis mit anschließender prolongierter Gabe. Zu beachten ist, dass die Dosierung, insbesondere bei hoher minimaler Hemmkonzentration (MHK) des Erregers oder erhöhter Antibiotika-Clearance (z. B. Augmented Renal Clearance – ARC, glomeruläre Filtrationsrate – GFR > 130 ml/min), idealerweise unter regelmäßigen und zeitnahen Blutspiegelkontrollen (Ergebnismitteilung Therapeutisches Arzneimittelmonitoring – TDM ≤ 24 h) individuell angepasst wird.

TDM ermöglicht bei Substanzen mit enger therapeutischer Breite (z. B. Vancomycin, Aminoglykoside) oder bei veränderter Nierenfunktion, Adipositas und schwer zugänglichen Infektionsherden (z. B. ZNS) eine individualisierte Dosierung. Der klinische Nutzen einer routinemäßigen Dosierungssteuerung über TDM ist bei kritisch kranken Patienten noch nicht abschließend belegt. Eine Metaanalyse zeigte eine signifikante Reduktion von Therapieversagen und Nephrotoxizität, jedoch ohne signifikante Senkung der Letalität [80]. Die Durchführung und Interpretation von TDM-Ergebnissen erfordern fundierte Kenntnisse der PK/PD-Eigenschaften und interdisziplinäre Zusammenarbeit zwischen Klinikern, Infektiologie, klinischer Pharmazie und Mikrobiologie. Alternativ können digitale Dosisberechnungstools oder evidenzbasierte Datenbanken^1^ genutzt werden.

### Therapiedauer.

Neuere Studien zeigen für viele Infektionen, einschließlich Bakteriämien, dass kürzere Behandlungszeiträume ebenso effektiv sind wie längere. Retrospektive Analysen zu Bakteriämien mit resistenten Extended-Spectrum-Beta-Lactamase(ESBL)-produzierenden [81] und Carbapenemase-produzierenden [82] Gram-negativen Erregern zeigten keine Unterlegenheit kurzer Therapiedauer mit < 7 Tagen bzw. < 10 Tagen.

Die Therapiedauer für die *S.-aureus*-Bakteriämie (SAB) ist lang. Methicillin-resistenter *Staphylococcus aureus* (MRSA) stellt einen Risikofaktor für eine prolongierte Bakteriämiedauer bei SAB dar [83]. Dies führt dazu, dass die SAB als kompliziert eingestuft und länger behandelt wird. Bisher gibt es jedoch keinen Hinweis darauf, dass der Nachweis von MRSA per se eine verlängerte Therapiedauer rechtfertigt [84, 85].

Auch für Vancomycin-resistente-Enterokokken-(VRE-)Bakteriämien liegen Daten vor: Eine retrospektive Studie verglich kurze (< 9 Tage) mit langen (> 9 Tage) Therapiedauern und zeigte ebenfalls kein schlechteres Ergebnis bei der kürzeren Behandlungsdauer [86].

Die aktuelle Evidenz spricht dafür, dass Infektionen mit MRE bei verfügbarer wirksamer Substanz keine grundsätzlich längere Behandlungsdauer erfordern. Diese Schlussfolgerung ist aufgrund der begrenzten Datenlage mit Vorsicht zu interpretieren.

### Intravenös-orale Sequenztherapie.

Die Sequenztherapie bietet viele Vorteile. In den letzten Jahren sind qualitativ hochwertige Studien zu dieser Fragestellung vorangetrieben worden und zeigen vielversprechende Ergebnisse [87]. MRE sind allerdings darin jedoch meist nur zu kleinen Anteilen eingeschlossen. Eine aktuelle Studie untersuchte die orale Sequenztherapie bei der Gram-negativen Bakteriämie [88]. ESBL-produzierende Erreger waren zu 16 % ursächlich diagnostiziert worden (20 % unter intravenöser Therapie, 13 % nach Oralisierung), eine Carbapenem-Resistenz wurde nur in 2 Fällen isoliert. Auch bei resistenten Erregern zeigte sich kein erhöhtes Therapieversagen unter Oralisierung, wenngleich die Gesamttherapiedauer insgesamt eher lang war.

Für die orale Sequenztherapie von MRSA steht Linezolid zur Verfügung, eine Substanz, die nach oraler Verabreichung fast vollständig resorbiert wird. Alternativ kommen ggf. auch Cotrimoxazol, Doxycyclin, Clindamycin oder Moxifloxacin infrage. Eine retrospektive Kohortenstudie untersuchte diese Therapie bei MRSA-Bakteriämie [89]. Dabei zeigte sich, dass die orale Linezolid-Therapie, die insbesondere bei unkomplizierten MRSA-Bakteriämien eingesetzt wurde, der parentalen Standardtherapie mit Vancomycin bzw. Daptomycin nicht unterlegen war. Inzwischen liegen 2 prospektive Studien zur Oralisierung bei unkomplizierter SAB vor [90, 91]. In der spanischen Studie wurde erfolgreich auf Linezolid oralisiert; im Oralisierungsarm lag der Anteil von MRSA bei 11 %. In der SABATO-Studie wurde für die Patienten mit nachgewiesenem MRSA (10 % im Oralisierungsarm) entweder Cotrimoxazol oder Linezolid verwendet, ebenfalls mit gutem klinischen Ergebnis. Auch für VRE-Infektionen steht Linezolid als Oralisierungsoption zur Verfügung. Systematische Studien für diesen Erreger fehlen jedoch bisher.

Insgesamt deutet die aktuelle Evidenz darauf hin, dass eine orale Sequenztherapie auch bei Infektionen mit resistenten Erregern möglich ist. Entscheidend ist dabei weniger die Resistenzlage selbst, sondern vielmehr, ob eine wirksame, oral gut bioverfügbare Substanz gegen den jeweiligen Erreger zur Verfügung steht – insbesondere im Bereich der Gram-negativen Erreger.

### Informationstechnologie.

In den letzten Jahren haben die Forschung zum Nutzen und die Implementierung klinischer Entscheidungsunterstützungssysteme wie Clinical Decision Support Systems (CDSS) und Computerized Physician Order Entry (CPOE) zugenommen [92]. Systematische Übersichtsarbeiten bestätigen, dass digitale Interventionen den Antibiotikaeinsatz reduzieren und dessen Angemessenheit verbessern können, während Effekte auf klinische Endpunkte (Sterblichkeit, Aufenthaltsdauer) widersprüchlich sind [93–95].

In der Cluster-randomisierten COMPASS-Studie in Krankenhäusern der Schweiz erhöhte eine multimodale CDSS-Intervention die angemessene Umstellung von intravenöser auf orale Verabreichung, verfehlte jedoch bei geringer CDSS-Nutzung das Ziel der Verbrauchsreduktion und unterstrich die Bedeutung von Akzeptanz und Integration in die Prozesse und Arbeitsabläufe [96].

In den INSPIRE-Studien führte die Implementierung von CPOE-Prompts, die patientenspezifische Risikoeinschätzungen für Infektionen mit MRE bereitstellten, zu einer signifikanten Reduktion des Einsatzes empirischer Breitspektrumantibiotika bei hospitalisierten Erwachsenen mit Pneumonie (−28 %), abdominalen (−35 %) und Haut-/Weichteilinfektionen (−23 %) ohne negative Auswirkungen auf klinische Ergebnisse wie Sterblichkeit, Verlegungen auf Intensivstationen oder Verweildauer zu haben. Die CPOE-Prompts empfahlen bei niedrigem MRE-Risiko (< 10 %) gezielt Schmalspektrum- anstelle von Breitspektrumantibiotika während der ersten 3 Krankenhaustage [97–99].

Multizentrische Studien in nichtuniversitären Krankenhäusern bestätigen, dass ABS-Programme auch in Deutschland zukünftig durch CPOE/CDSS unterstützt werden können [100]. Die berichteten Erfolge erfordern jedoch kontinuierliche Ressourcen (z. B. Datenpflege), Interoperabilität der Systeme, Anwenderfreundlichkeit und Personal [100, 101]. Die Integration von CDSS-Systemen bis hin zur Entwicklung von Machine-Learning-Techniken kann in Zukunft das Erreichen der Ziele von ABS möglicherweise vereinfachen [102].

## Fazit

In Deutschland haben unterschiedlichste ABS-Aktivitäten, basierend auf Eigeninitiativen oder im Rahmen zeitlich begrenzter Projektförderungen, in der ambulanten und stationären Versorgung insbesondere zur Reduktion der Verordnungsintensität in der Humanmedizin beigetragen. Optimierungspotenzial besteht seit vielen Jahren in der adäquaten Indikationsstellung, der gezielten und unterstützenden Nutzung von Infektionsdiagnostik und elektronischen Verordnungssystemen. Hierfür sind Konzepte zur dauerhaften Finanzierung der einzubringenden infektionsmedizinischen Fachexpertise, abgesichert durch politische Unterstützung, erforderlich.

### Infobox Regionale/lokale Antibiotic-Stewardship(ABS)-Netzwerke in Deutschland. Quelle: Akademie für Infektionsmedizin: Regionale Netzwerke^a^

- ABS-Netzwerk Nord (nur Kliniken)
- ABS-Netzwerk Osnabrück/West-Niedersachsen (nur Kliniken)
- ABS-Netzwerk Westfalen-Lippe, mit ABS-Netzwerk Bielefeld/Ostwestfalen-Lippe und AnTiB
- ABS-Netzwerk West (nur Kliniken)
- AMS-Netzwerk Mainfranken (nur Kliniken)
- ABS-Netzwerk Rhein-Neckar (nur Kliniken)
- ABS-Netzwerk München (Kliniken + Öffentlicher Gesundheitsdienst (ÖGD))

^a^ https://www.akademie-infektionsmedizin.de/abs/netzwerk/regionale-netzwerke/

## Supplementary Information

Onlinematerial: Tab. S1. Vergleich ausgewählter molekularer und phänotypischer Systeme zur beschleunigten Identifikation und Empfindlichkeitsprüfung von Erregern aus Blutkulturen

## Footnotes

1 Beispiele für digitale Dosisberechnungstools bzw. -datenbanken: www.thecaddy.de, www.doseeasy.de, www.dosing.de.
