# Supplementary material for: Requirements for the rational use of antibiotics based on antibiotic stewardship
Source: Bundesgesundheitsblatt Gesundheitsforschung Gesundheitsschutz. 2026 Mar 30;69(5):556–66. [Article in German] doi: 10.1007/s00103-026-04224-8 (PMC13133214; doi:10.1007/s00103-026-04224-8)
Supplement: Supplementary file 1 — Onlinematerial: Tab. S1. Vergleich ausgewählter molekularer und phänotypischer Systeme zur beschleunigten Identifikation und Empfindlichkeitsprüfung von Erregern aus Blutkulturen [file 103_2026_4224_MOESM1_ESM.pdf]

Tab. S1. Vergleich ausgewählter molekularer und phänotypischer Systeme zur beschleunigten Identifikation und Empfindlichkeitsprüfung von Erregern aus Blutkulturen. Abkürzungen: AST=*antibiotic susceptibility tesing (*dt. Empfindlichkeitstestung); FISH=Fluoreszenz-in-situ-Hybridisierung; LAMP=*loop-mediated isothermal amplification* (dt. isothermes DNA-Amplifikationsverfahren). Min.=Minuten, Std.=Stunden

System	Hersteller	Technologie / Prinzip	Laufzeit[a]	Nachweisumfang	Literatur[b]
molekulare/kommerzielle Systeme					
BioFire FilmArray BCID / BCID2	bioMérieux (Frankreich)	Multiplex-PCR (all in one)	60–75 Min.	Bis zu 43 Erreger + 10 Resistenzgene (*mecA*, *bla*$_{CTX-M}$, *vanA/B*)	Berinson et al. (2012); Schumann et al. (2021); Chen et al. (2013)
Verigene BC-GN / BC-GP	Luminex Corporation (USA)	Microarray-basierte Nukleinsäurehybridisierung	2–3 Std.	Gram-positive bzw. Gram-negative Erreger + Resistenzgene	Arroyo et al. (2017); Claeys et al. (2021); Walker et al. (2016)
GenMark ePlex BCID Panel	Roche Diagnostics (Schweiz)	Cartridge-basiertes Multiplex-PCR-System	90 Min.	Ca. 30 Pathogene + Resistenzdetektion	Caspar et al. (2024); Kramme et al. (2025); Wolk et al. (2021)
T2Bacteria / T2Candida Panels	T2 Biosystems (USA)	T2-Magnetresonanz-Assay (direkt aus Vollblut)	3–5 Std.	Häufigste Sepsis-Erreger direkt aus Blut (Erregerspektrum: *E. faecium, S. aureus, K. pneumoniae, A. baumannii, P. aeruginosa, and E. coli* and *C. albicans*/*C. tropicalis*, *C. krusei/C. glabrata*, and *C. parapsilosis*)	De Angelis et al. (2018); Lucignano et al. (2022)
Accelerate Pheno System	Accelerate Diagnostics (USA)	FISH-basierte Identifikation + mikroskopischer phänotypischer AST	5–7 Std.	Erregeridentifikation + Empfindlichkeitsprofil	Kramme et al. (2025); Chapot et al. (2021); Dare et al. (2021)
eazyplex System	Amplex Diagnostics (Deutschland)	Isothermale LAMP-Amplifikation	15–30 Min.	Pathogene & ausgewählte Resistenzgene (*mecA*, *bla*$_{KPC}$, *bla*$_{NDM}$, *bla*$_{OXA-48-like}$, *vanA/B*)	Bach et al. (2022); Boattini et al. (2025)

Kulturbasierte Methoden						
	Qvella FAST-System	Qvella Corporation (Kanada)	Direktnachweis (Nukleinsäure-Extraktion + PCR-ready)	2-3 Std.	Schnellisolation für nachgeschaltete PCR oder direkte MALDI-TOF	Penven et al. (2025); Sy et al. (2023)
	MALDI-TOF MS	Bruker Daltonics / bioMérieux	Massenspektrometrie-basierte Proteinprofilanalyse zur Erregeridentifikation Direkt aus der positiven Blutkulturflasche	3-4 Std.	Identifikation von Bakterien, Hefen und einigen Mykobakterien aus Kulturmaterial bzw. aus positiver Blutkultur nach Aufbereitung.	Chen et al. (2013); Buchan et al. (2012); Klein et al. (2012)
-	EUCAST-RAST + MALDI-TOF MS ID	European Committee on Antimicrobial Susceptibility Testing (EUCAST)	Disk-Diffusion-basiertes Resistenztestung und Speziesidentifikation über Massenspektrometrie	4–6 Std.	Beschleunigte phänotypische Empfindlichkeitstestung und Speziesidentifizierung aus positiver Blutkultur	Cherkaoui et al. (2022); Jasuja et al. (2021)

[a] Zeitdauer nach der initialen Bebrütung der Blutkulturflaschen im Blutkulturautomaten.
[b] ausgewählte Referenzen
